# Computational Biology: Modeling Chronic Renal Allograft Injury

**DOI:** 10.3389/fimmu.2015.00385

**Published:** 2015-08-03

**Authors:** Mark D. Stegall, Richard Borrows

**Affiliations:** ^1^Department of Surgery, von Liebig Transplant Center, Mayo Clinic, Rochester, MN, USA; ^2^Queen Elizabeth Hospital Centre, Nephrology, Birmingham, UK

**Keywords:** renal transplantation, computational biology, chronic renal allograft dysfunction, immunology, mathematical modeling

## Abstract

New approaches are needed to develop more effective interventions to prevent long-term rejection of organ allografts. Computational biology provides a powerful tool to assess the large amount of complex data that is generated in longitudinal studies in this area. This manuscript outlines how our two groups are using mathematical modeling to analyze predictors of graft loss using both clinical and experimental data and how we plan to expand this approach to investigate specific mechanisms of chronic renal allograft injury.

## Introduction

Improving long-term renal allograft survival is one of the major unmet needs in organ transplantation. It is a sad fact that the rate of late graft loss (2–3%/year beyond the first year) appears to have changed little over the past two decades (1). While some progress has been made in understanding the multiple causes of late renal allograft loss, our picture is still incomplete (2, 3).

The goal of this manuscript is to outline how our two groups have already started to use mathematical modeling to analyze predictors of graft loss using both conventional clinical data and more detailed histologic and genomic data. We also outline how we plan to expand this approach going forward to investigate specific mechanisms of progressive injury.

## Complexity of Transplant Outcomes

Post-transplant events are maddeningly complex. All renal allografts are exposed to at least one type of injury-causing process and most are exposed to several. Yet, the vast majority of grafts function quite well for many years. Serial surveillance biopsies suggest that several pathologic processes may lead to chronic injury ultimately resulting in graft loss (3). Importantly, these studies suggest that the process may be present for years before a clinically significant endpoint is reached and patients who seem to have similar pathologic processes may have very different outcomes. Some progress to graft loss, some develop chronic injury yet maintain function, and still others appear to have no injury. Subclinical inflammation and chronic antibody-mediated rejection due to donor-specific alloantibody (DSA) are two good examples. A remarkable calculus exists in which multiple different pathologic influences, occurring with varying frequency and severity at different time points, result in an almost linear rate of graft loss over many years in the entire population.

It is important to identify grafts that will fail at an early time point when the graft function is good and thus salvageable. Since not all grafts with DSA or subclinical inflammation will fail, it is also important to determine which features of the chronic immunologic injury process predispose to graft failure and thus develop specific therapy for progressors.

Our understanding of the mechanisms by which a biological process takes years to reach a clinically significant endpoint is lacking. However, mathematical modeling of increasingly comprehensive and complex data appears to be a promising path forward.

## Modeling Renal Allograft Loss Using Clinical Factors

Mathematical models that aim to predict renal allograft outcomes based on clinical factors have been around for years. In the United States, the Federal Government through the Scientific Registry of Transplant Recipients issues “center-specific” expected outcomes for patient and graft survival based on a combination of donor and recipient factors present pretransplant (4). Combined, the *C*-statistic for this model is estimated to be only 0.6 (5). Two of the most important recipient factors affecting outcomes that are present pretransplant are age and diabetes.

However, renal transplantation is a dynamic process, and post-transplant events clearly affect outcomes. Several groups have tried to develop outcomes models based on post-transplant factors. One such model from Birmingham, UK, uses factors present at 1-year post-transplantation to predict graft survival at 5 years (6). The factors that go into the predictive formula are both demographic data and clinical data points present at 1 year including estimated glomerular filtration rate at 1 year, age at 1 year, recipient race, sex, presence of absence of rejection at 1 year, urinary albumin to creatinine ratio at 1 year, and serum albumin at 1 year. Risk scores were generated based on calculations of weighted coefficients from the regression analyses.

This “Birmingham Model” was validated in four independent cohorts from three other centers (Tours, France; Leeds, United Kingdom; and Halifax, Canada). It showed good discrimination for both overall graft failure (*C* statistics 0.75–0.81) and for death-censored graft failure (*C* statistics 0.78–0.90). Discrimination alone is insufficient to determine the utility of a risk model. Therefore, other measures were evaluated in the cohorts described above, specifically calibration (a comparison of rates of expected and observed outcomes across risk strata) and risk reclassification [evaluation of incremental accuracy of the model above and beyond accepted and existing measures, in this case renal function (eGFR)]. The “Birmingham Risk Score” similarly performed well across these domains.

However, other potentially important biological data were lacking from these studied datasets. Notably, histological data (specifically protocol biopsy findings at the 1-year time point post-transplantation) were not analyzed and anti-HLA antibodies (“alloantibody”) tested simultaneously were not evaluated. These potential “predictors” have much in common: they are both emerging risk factors for outcome, but are not yet universally incorporated into clinical practice; they require specialist analysis, which is time-consuming, labor-intensive, and expensive; the results require careful evaluation alongside clinical data; the results may be bewilderingly complex with a single “analysis” yielding multiple outputs, which may or may not be interdependent. It is for the former reasons that many centers do not collect these data, and it is for the latter reasons that detailed mathematical and computational modeling is vital to understand their relevance.

## Adding Histology and Alloantibody Data to Predictive Models

**Figure 1:**
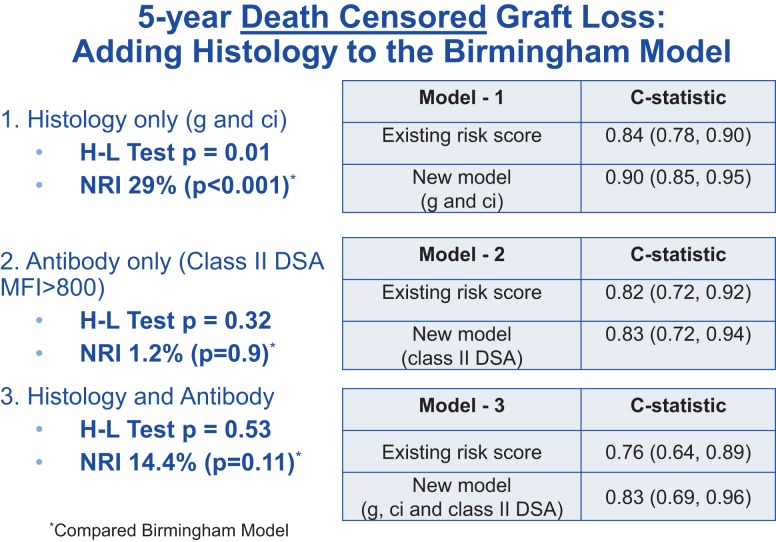
**Data showing that the risk score *C*-statistic is improved by adding histology and calibration improves by adding antibody**.

Histologic findings at 1 year have been shown to correlate with outcomes (7). In a recent collaborative study between the Birmingham group and the Mayo Clinic, Rochester, MN, USA, the Birmingham model was again validated in a Mayo Clinic population consisting primarily of living donor kidney transplants (8). In the Mayo cohort, the presence of glomerulitis (g) and chronic interstitial fibrosis (ci) found on 1 year protocol biopsy independently predicted 5-year graft failure. The presence of anti-class II donor-specific antibody (DSA) in the serum 1 year post-transplantation was also associated with adverse outcome. When a new prognostic model was developed by incorporating these standard histological qualifiers (by conventional light microscopy) alongside other clinical variables, discrimination (compared with the original Birmingham risk Score) was improved, with the *C*-statistic increasing from 0.84 to 0.90 (Figure 1). The stepwise addition of DSA data did not further improve discrimination, presumably because the presence of alloantibody-associated histological injury already “captured” the antibody effect. Furthermore, the new risk model improved calibration and (again, in comparison with the original model) resulted in statistically significant and clinically relevant risk reclassification with a net reclassification improvement (“NRI”) of 29% for the endpoint of death-censored graft survival (*p* = 0.01). The inclusion of both histology and antibody also resulted in improved reclassification of outcome, although with borderline statistical significance (*p* = 0.11).

## Mathematical Modeling: A Method to Identify *Mechanisms* of Chronic Injury?

In reality, clinical factors such as age and race are simply surrogates for biological processes that cause graft loss. Similarly, non-specific laboratory findings, such as renal function and proteinuria, although good readouts for damage, do not provide detailed insight into the actual mechanisms of renal allograft injury. As we move further down the pathway from non-specific data to more detailed data, we likely will not only reach higher levels of prediction but also begin to understand the underlying mechanisms of progressive injury. Using the approach outlined above, any type of molecular, histologic, or serologic data can be examined in mathematical models to determine its effect on outcome.

## Molecular Signatures and Other Biomarkers

The past several years have seen the development of novel biomarkers and it is possible that the inclusion of some of these variables might further improve our ability to predict graft outcome. They might also improve our ability to diagnose specific pathologic processes and design intervention studies. These other approaches include gene expression and proteomic profiles in the graft, peripheral blood, or urine; more detailed DSA characterization, such as C1q binding; and/or more detailed histologic studies including immunohistochemistry for specific cell types of the infiltrates. Of these, “omics” studies deserve special mention here (9–19).

Gene expression signatures correlating with acute cellular rejection have been identified in peripheral blood and they are on their way to becoming clinically-available tests (13, 17). A signature has been identified in renal allograft biopsies that correlates with antibody-mediated rejection (9, 15). Other signatures have been identified that correlate with patients who are “operationally tolerant” (i.e., off immunosuppression and have stable kidney or liver allograft function). In addition, microRNA signatures have been correlated with rejection (18) and diabetic nephropathy (19).

It is likely that some of these molecular signatures also might be shown to correlate with late graft outcomes, but how well they actually predict graft loss is unclear. Currently, these primary value of these tests is that they appear to correlate well with known histologic findings and thus in some cases they may obviate the need for surveillance biopsies. Currently, none of these novel signatures is being used to identify progression of injury or is used in a model of chronic injury similar to the Birmingham model.

Another possible use of these omics data is that of a biomarker that would serve as a surrogate endpoint for clinical trials aimed at improving long-term graft survival. Under “accelerated approval,” the FDA might approve a drug based on its improving surrogate makers at an early time point (20). Longer-follow up would then be continued in Phase 3 studies to confirm improvement in the true clinical endpoint, such as graft survival. Thus, modeling the mechanisms of long-term graft survival will be important in the development of new therapeutics. Unfortunately, the development of effective biomarkers has been difficult in almost all fields of medicine and we must proceed down this pathway with some caution (21).

## Other Mathematical Model Issues

There are several causes of late graft loss and each may require a different therapeutic approach. Thus, identifying specific subtypes of patients with a specific known injury process and then modeling what aspects of that process are involved in progression will be an important path toward new therapy. One of the most important issues to consider when we begin to concentrate on subtypes of chronic injury is the issue of patient classification. Clearly identifying the phenotype categories will be important. We are unlikely to find the cause of progression in patients with alloantibody at 1 year if they are lumped together with all patients with low renal function at 1 year. Indeed, computational methods might be able to identify the phenotypes.

Another issue is dealing with several factors that are all part of the same process. For example, in the histologic study mentioned above, the inclusion of glomerulitis (a process associated with alloantibody) probably obviated the need to include DSA in order to improve the discrimination of the model. However, this does not mean that DSA is unimportant in chronic injury, and in fact is likely to be a major mechanistic driver. Underpowered studies also can lead to false-negative assessment in modeling and must be considered. There are far more “null” studies in transplantation than truly “negative” ones, and although the latter may inform practice, the former require recognition and refinement.

Mathematical modeling may identify the presence of processes for which we have no data. In the case of DSA, there is experimental data suggesting that an allograft may develop resistance of DSA, termed accommodation (22–24). Mathematically, this might be viewed as a “vector” that would favor graft survival even when erstwhile injury-causing stimuli are present. We then would be charged with searching for processes that might explain the observed outcomes. Sir Arthur Eddington might understand this (25).

Finally, when considering a process that occurs over many years, it is likely that other injury-causing events might also occur. For example, in the renal allograft setting, chronic hypertension, diabetes, nephrotoxicity from calcineurin inhibitors, and recurrent disease are just a few of the many possible injury stimuli that might also be occurring in addition to immunologic injury. Mathematical modeling also will likely able to control for all of the different injury processes present in the graft. It is likely that there will be common features and, hopefully, specific features. Separating other causes of injury from the primary process being studied adds yet another complicating factor in this type of research.

## How to Optimize Mathematical Modeling in Transplantation?

We contend that a major impact of computational biology will be to enhance our ability to study chronic renal allograft injury in humans (Table 1). The critical component for these studies will be data –lots and lots of detailed, accurate data. Data regarding the recipient’s immune response and biomarkers that are related to the pathologic process under study would be helpful. Mechanistic studies done in parallel to focused clinical trials also would be tremendously useful. For example, combining gene expression studies with a trial of an agent that specifically blocks one pathway (e.g., eculizumab or IL-6 receptor) might provide new insight into why grafts fail.

**Table 1:** **Possible approaches to using computational biology to studying chronic renal allograft injury**.

∙ Comprehensive assessment of subjects
∘ Immune system assays
∘ Target tissue assessment
∙ Long-term studies with serial assessments
∙ Biomarkers related to the biology/targeted interventions
∘ Omics studies of peripheral blood lymphocytes, serum, plasma, urine, or tissue
∘ Detailed alloantibody studies

We also need detailed long-term data beyond what is currently available. Graft survival at 5 years is just the beginning. What happens between 5 and 10 years? We need to model these later time points and this will require data that are rarely captured.

In most disease groups, there are many phenotypes and small numbers of patients in each phenotype. Thus, in order to study sufficient numbers of patients, these studies will need to be multicenter and very collaborative and we may need to combine data from many different databases.

Finally, studying a complex biologic process, such as chronic injury, probably will require a change in mindset among researchers. We tend to strive to make model systems as simple as possible with as few variables. While this makes for good science, it may be an inadequate approach to studying chronic injury.

## Summary

The application of computational biology to transplantation seems to be a natural progression of both fields. The interaction between mathematicians and transplant biologists will likely lead to novel new interpretations of phenomena and new understanding of the mechanisms of chronic injury.

## Conflict of Interest Statement

Mark D. Stegall has research contracts or consulting with Alexion, Astellas, Millennium, True North, Immunocor, and Genentech. Richard Borrows declares no conflict of interest.

## References

[1] Lamb KE Lodhi S Meier-Kriesche H-U. Long-term renal allograft survival in the United States: a critical reappraisal. Am J Transplant (2011) 11:450–62. 10.1111/j.1600-6143.2010.03283.x 20973913

[2] El-Zoghby ZM Stegall MD Lager DJ Kremers WK Amer H Gloor JM Identifying specific causes of kidney allograft loss. Am J Transplant (2009) 9:527–35. 10.1111/j.1600-6143.2008.02519.x 19191769

[3] Stegall MD Gaston RS Cosio FG Matas A. Through a glass darkly: seeking clarity in preventing late kidney transplant failure. J Am Soc Nephrol (2015) 26:20–9. 10.1681/ASN.2014040378 25097209 PMC4279746

[4] Available from: http://www.srtr.org/csr/current/Centers/201412/all_csr_documentation.pdf

[5] Wolfe RA McCullough KP Schaubel DE Kalbfleisch JD Murray S Stegall MD Calculating life years from transplant (LYFT): methods for kidney and kidney-pancreas candidates. Am J Transplant (2008) 8:997–1011. 10.1111/j.1600-6143.2008.02177.x 18336702

[6] Shabir S Halimi JM Cherukuri A Ball S Ferro C Lipkin G Predicting 5-year risk of kidney transplant failure: a prediction instrument using data available at 1 year posttransplantation. Am J Kidney Dis (2014) 4:643–51. 10.1053/j.ajkd.2013.10.059 24387794

[7] Cosio FG Grande JP Wadei H Larson TS Griffin MD Stegall MD. Predicting subsequent decline in kidney allograft function from early surveillance biopsies. Am J Transplant (2005) 5:2464–72. 10.1111/j.1600-6143.2005.01050.x 16162196

[8] Moreno-Gonzalez M Bentall A Borrows R Stegall MD Predicting 5-year risk of kidney transplant failure: does histology add to clinical parameters available at 1 year post-transplantation? Am J Transplant (2015) 15:1 10.1111/ajt.13359

[9] Sis B Jhangri GS Bunnag S Allanach K Kaplan B Halloran PF. Endothelial gene expression in kidney transplants with alloantibody indicates antibody-mediated damage despite lack of C4d staining. Am J Transplant (2009) 9:2312–23. 10.1111/j.1600-6143.2009.02761.x 19681822

[10] Park WD Griffin MD Cornell LD Cosio FG Stegall MD. Fibrosis with inflammation at one year predicts transplant functional decline. J Am Soc Nephrol (2010) 21:1987–97. 10.1681/ASN.2010010049 20813870 PMC3014013

[11] Newell KA Asare A Kirk AD Gisler TD Courcier K Suthanthiran M Identification of a B cell signature associated with renal transplant tolerance in humans. J Clin Invest (2010) 120:1836–47. 10.1172/JCI39933 20501946 PMC2877933

[12] Baron D Ramstein G Chesneau M Echasseriau Y Pallier A Paul C A common gene signature across multiple studies relate to biomarkers and functional regulation in tolerant to renal allograft. Kidney Int (2015) 87:984–95. 10.1038/ki.2014.395 25629549 PMC4424816

[13] Kurian SM Williams AN Gelbart T Campbell D Mondala TS Head SR Molecular classifiers for acute kidney transplant rejection in peripheral blood by whole genome gene expression profiling. Am J Transplant (2014) 14:1164–72. 10.1111/ajt.12671 24725967 PMC4439107

[14] Kurian SM Fouraschen SM Langfelder P Horvath S Shaked A Salomon DR Genomic profiles and predictors of early allograft dysfunction after human liver transplantation. Am J Transplant (2015) 15:1605–14. 10.1111/ajt.13145 25828101

[15] Loupy A Lefaucheur C Vernerey D Chang J Hidalgo LG Beuscart T Molecular microscope strategy to improve risk stratification in early antibody-mediated kidney allograft rejection. J Am Soc Nephrol (2014) 25:2267–77. 10.1681/ASN.2013111149 24700874 PMC4178445

[16] Roedder S Li L Alonso MN Hsieh SC Vu MT Dai H A three-gene assay for monitoring immune quiescence in kidney transplantation. J Am Soc Nephrol (2014). 10.1681/ASN.2013111239 25429124 PMC4520154

[17] Roedder S Sigdal T Salamonis N Hsieh S Dai H Bestard O The kSORT assay to detect renal transplant patients at high risk for acute rejection: results of the multicenter AART study. PLoS Med (2014) 11:e1001759 10.1371/journal.pmed.1001759 25386950 PMC4227654

[18] Anglicheau D Sharma VK Ding R Hummel A Snopkowski C Dadhania D MicroRNA expression profiles predictive of human renal allograft status. Proc Nat Acad Sci U S A (2009) 106:5330–5. 10.1073/pnas.0813121106 19289845 PMC2663998

[19] Bijkerk R Duijs JM Khairoun M Ter Horst CJ van der Pol P Mallat MJ Circulating micoRNAs associate with diabetic nephropathy and systemic microvascular damage and normalize after simultaneous pancreas-kidney transplantation. Am J Transplant (2015) 15:1081–90. 10.1111/ajt.13072 25716422

[20] Subpart H Accelerate Approval for New Drugs for Serious or Life-Threatening Illnesses 20xx. Available from: http://www.accessdata.fda.gov/scripts/cdrh/cfdocs/cfcfr/CFRSearch.cfm?CFRPart=314&showFR=1&subpartNode=21:5.0.1.1.4.8

[21] Ioannidis JPA Biomarkers failures. Clin Chem (2013) 59:202–4. 10.1373/clinchem.2012.185801 22997282

[22] Koch CA Khalpey ZI Platt JL. Accommodation: preventing injury in transplantation and disease. J Immunol (2004) 172:5143–8. 10.4049/jimmunol.172.9.5143 15100249

[23] Chen Song S Zhong S Xiang Y Li JH Guo H Wang WY Complement inhibition enables renal allograft accommodation and long-term engraftment in presensitized nonhuman primates. Am J Transplant (2011) 11:2057–66. 10.1111/j.1600-6143.2011.03646.x 21831160

[24] Dorling A. Transplant accommodation – are the lessons learned from xenotransplantation pertinent for clinical allotransplantation? Am J Transplant (2012) 12:545–53. 10.1111/j.1600-6143.2011.03821.x 22050724

[25] Available from: http://www.wired.com/2009/05/dayintech_0529/

